# The Orienting Response in Healthy Aging: Novelty P3 Indicates No General Decline but Reduced Efficacy for Fast Stimulation Rates

**DOI:** 10.3389/fpsyg.2017.01780

**Published:** 2017-10-17

**Authors:** Stefan Berti, Gerhard Vossel, Matthias Gamer

**Affiliations:** ^1^Department of Psychology, Johannes Gutenberg-University Mainz, Mainz, Germany; ^2^Department of Psychology, University of Würzburg, Würzburg, Germany

**Keywords:** attention, change detection, auditory system, novelty processing, event-related potential (ERP), P300, skin conductance response (SCR)

## Abstract

Automatic orienting to unexpected changes in the environment is a pre-requisite for adaptive behavior. One prominent mechanism of automatic attentional control is the Orienting Response (OR). Despite the fundamental significance of the OR in everyday life, only little is known about how the OR is affected by healthy aging. We tested this question in two age groups (19–38 and 55–72 years) and measured skin-conductance responses (SCRs) and event-related brain potentials (ERPs) to novels (i.e., short environmental sounds presented only once in the experiment; 10% of the trials) compared to standard sounds (600 Hz sinusoidal tones with 200 ms duration; 90% of the trials). Novel and standard stimuli were presented in four conditions differing in the inter-stimulus interval (ISI) with a mean ISI of either 10, 3, 1, or 0.5 s (blocked presentation). In both age groups, pronounced SCRs were elicited by novels in the 10 s ISI condition, suggesting the elicitation of stable ORs. These effects were accompanied by pronounced N1 and frontal P3 amplitudes in the ERP, suggesting that automatic novelty processing and orientation of attention are effective in both age groups. Furthermore, the SCR and ERP effects declined with decreasing ISI length. In addition, differences between the two groups were observable with the fastest presentation rates (i.e., 1 and 0.5 s ISI length). The most prominent difference was a shift of the peak of the frontal positivity from around 300 to 200 ms in the 19–38 years group while in the 55–72 years group the amplitude of the frontal P3 decreased linearly with decreasing ISI length. Taken together, this pattern of results does not suggest a general decline in processing efficacy with healthy aging. At least with very rare changes (here, the novels in the 10 s ISI condition) the OR is as effective in healthy older adults as in younger adults. With faster presentation rates, however, the efficacy of the OR decreases. This seems to result in a switch from novelty to deviant processing in younger adults, but less so in the group of older adults.

## Introduction

The Orienting Response (OR) is a fundamental mechanism, which enables the automatic detection of and orienting to unexpected events or changes in the environment (see Pavlov, 1927; Sokolov, 1963, 1990; Lynn, 1966). The OR is described as a whole-body phenomenon (Sokolov, 1963; Lynn, 1966; see also Barry, 2009) because the processing of a sudden and unexpected event in the environment results in effects on different levels of bodily responses (i.e., motor system, autonomic nervous system, and central nervous system). Therefore, the OR is best tapped by combined measures of different physiological parameters. In contrast to the fundamental importance of this response, effects of healthy aging on the OR are rarely investigated. In the present study, we examined the OR in two different age groups (19–38 years and 55–72 years) and tested whether healthy aging affects the central nervous system response of this fundamental mechanism.

### Automatic change detection and the orienting response (OR)

The theory of the OR describes it as a fundamental pre-requisite for adaptive behavior in constantly changing environments. In short, the OR manifests an immediate response to changes in the environment on a physiological, behavioral, and cognitive level (Lynn, 1966). A variety of sensory stimuli can elicit an OR in everyday life including such different stimuli like one's own name when sitting in a lecture hall or a suddenly approaching object in traffic. Typically, stimuli that are unpredictable, rare, and significant are capable of eliciting an OR (see Lynn, 1966). In addition, too high stimulus intensity may result in defensive responding (e.g., startle reflex) while stimuli with a too low intensity will go unnoticed. These characteristics are sometimes summarized in the notion that the OR is a response to novelty (but see Velden, 1978; Bernstein, 1979).

The OR allows for flexible adaptive behavior to sudden and—presumably—unexpected changes in the environment, which indicate a potentially relevant or even dangerous situation. This implies that sensory change detection underlying the OR is an automatic, pre-attentive process. From a subjective point of view, the OR is perceived as a disruption of the current attentional focus (e.g., to a task at hand or a current goal). This involuntary attention switching is a feature that the OR shares with other mechanisms of (automatic) change detection, e.g., the detection of stimuli deviating from a continuous sensory auditory (i.e., auditory oddball) stimulation. The OR, however, also comprises autonomic physiological and motor effects: A typical OR increases the arousal level and triggers a bodily orientation to the source of the detected change. This response on different levels (i.e., arousal and motor response) constitutes a distinct feature of the OR because other processes of automatic change detection do not result in such a pattern of responses.

### Psychophysiological correlates of the OR

The OR is typically quantified with psychophysiological measures, namely electrodermal activity (EDA) and event-related brain potentials (ERPs). On the one hand, these measures are online measures of information processing and therefore allow to unravel the detailed time course of OR processing even in passive participants. On the other hand, EDA and ERP parameters tap into different aspects of the OR: In the EDA, event-related changes in skin conductance (i.e., skin conductance responses, SCR) mirror changes in the activity level of the sympathetic nervous system. In other words, the SCR is a correlate of the arousal level, which increases after an OR. In the ERP, the frontal portion of the P3 complex (a positive deflection in the ERP around 300 ms after event onset) mirrors the allocation or orientation of attention to the new object detected in the environment. In other words, the frontal P3 of the ERP mirrors the allocation of central cognitive resources to the detected change (see Friedman et al., 2001; Polich, 2007). With this, a combined application of the EDA and the ERP methodology allows for the identification and quantification of an OR in laboratory research.

ERPs, however, are also a valuable tool for investigating other forms of automatic change detection or involuntary orientation of attention. The most prominent line of research is related to the so-called Mismatch Negativity (MMN) of the ERP, which is typically investigated with oddball or oddball-like stimulation protocols. The classical oddball stimulation consists of two types of stimuli: a frequent standard stimulus and a rare deviant stimulus; in addition, the appearance of the deviation in the stimulation is not predictable by the listener. The MMN is a negative deflection peaking around 200 ms after the presentation of a deviant and is best mirrored in the difference waves between standard and deviant trials. One main characteristic of the MMN is that it is elicited irrespective of whether the participants are attending to the stream of presentation or become aware of the deviation. While this was extensively demonstrated in the auditory modality (see Näätänen et al., 2007), there is increasing evidence that the MMN also mirrors automatic and pre-attentive change detection in the visual modality (Berti, 2011; Flynn et al., 2017; Jack et al., 2017). The second main feature is that the MMN is often followed by a frontal P3 or P3a (for instance, when the deviants become task-relevant). This and other theoretical considerations lead to the interpretation that the MMN-related processes may trigger or contribute to the OR (see Näätänen, 1992).

In recent ERP-studies (see Rinne et al., 2006; Berti, 2012, 2013; Lange et al., 2015; Barceló and Cooper, 2017) such a unitary view of the mechanisms underlying automatic change detection in an auditory oddball paradigm was questioned. For instance, Rinne et al. (2006) demonstrated that the pattern of deviance-related ERP components differed between intensity increment and intensity decrement deviants. A study by Berti (2013) extended this view to two different mechanisms of change detection, one based on the automatic detection of deviancy within a classical oddball stimulation and one based on the detection of auditory transients (i.e., rare auditory stimuli which are not embedded within a stream of standard auditory stimuli). Furthermore, Berti (2013) argued that the typical pattern of ERP components related to auditory change detection as revealed in the standard oddball stimulation might be a mixture of different overlapping processes, namely deviance processing and transient processing with the latter related to the OR. Lange et al. (2015) tested a comparable hypothesis directly and demonstrated that within the P3 time window, different frontal P3 subcomponents could be separated which are related to different processes. In this study, Lange et al. (2015) separated an uncertainty P3 and a novelty P3 and concluded that these two functional subcomponents of the frontal P3 mirror different aspects of OR processing. The results by Berti (2013) and Lange et al. (2015) are in close accordance with the argumentation of Barry et al. (2016). In this study, the authors demonstrated that these presumably overlapping mechanisms can be separated in the P3 by means of temporal principal component analysis and that the classical novelty P3 as a correlate of the OR can be distinguished from other P3 subtypes. Taken together, these studies demonstrate (1) that the P3 is indeed an OR correlate but (2) that the identification of an OR by means of the P3 might be difficult because of overlapping effects of different change detection mechanisms. However, following the terminology suggested by Barry et al. (2016), we separate the frontal P3s into the earlier P3a and the later novelty P3 (nP3) and regard the nP3 as an OR correlate (see also Friedman et al., 2001).

### Aging effects on automatic change detection

Because automatic change detection is one of the basic neuro-cognitive mechanisms enabling fast and flexible behavioral adaptation to unexpected changes in the environment, effects of age on these functions have been in the focus of scientific research for many years. One central question is how the automatic processing of unexpected changes in the stimulation is affected by age. This perspective assumes that aging necessarily impairs sensory and cognitive effects (see Li and Lindenberger, 2002) and that sensory and cognitive processing in young adults (in the 3rd and 4th decade of life) depicts “normal” and healthy mechanisms of neuro-cognitive processing. In contrast, it is worth noting that especially the development of the neo-cortex over the life span exhibits different progressions in different areas (see Sowell et al., 2003), which does not support the idea of one ideal developmental stage in early adulthood. In other words, healthy aging may result in different forms of variations over the life span and based on increasing experience also gains of neuro-cognitive functioning are feasible.

The auditory oddball paradigm combined with ERP measures provides a direct approach for evaluating aging effects on automatic stimulus processing and attentional allocation. Consequently, several studies tested aging effects on the MMN as an indicator of effective pre-attentive change detection and the frontal P3/P3a as an indicator of involuntary orientation of attention. Several studies reported aging effects on both ERP components (e.g., Pekkonen et al., 1993; Fabiani and Friedman, 1995; Fabiani et al., 1998; Gaeta et al., 1998, 2001, 2002; Alain and Woods, 1999; Pekkonen, 2000; for reviews see Kok, 2000; Friedman, 2003; Alain et al., 2004; Ruzzoli et al., 2012). Regarding the MMN, typically a decline of the amplitude with age was reported, suggesting a decrease in the efficacy but no complete lack of pre-attentive, sensory processing (for a review see Pekkonen, 2000; Cooper et al., 2006; Rimmele et al., 2012). From this, Alain et al. (2004) argued that aging results in a shift from automatic to controlled processing of deviations in sensory stimuli. With regard to the P3a, Fabiani and colleagues (Fabiani and Friedman, 1995; Fabiani et al., 1998) also reported aging effects regarding variations in the distribution of the P3a peak from a frontal maximum in younger adults to more central scalp areas in older adults. Moreover, Rimmele et al. (2012) reported a lack of P3a in older adults to deviants within tone patterns. Taken together, this pattern of ERP results suggests that the efficacy of sensory pre-processing and involuntary allocation of attention declines in higher adulthood.

In contrast, some recent studies applying a variant of the oddball paradigm reported a different pattern of results (see Mager et al., 2005; Horváth et al., 2009; Berti et al., 2013; Getzmann et al., 2013; Correa-Jaraba et al., 2016). The main difference in these studies was that participants performed an active task but this task was not related to the rare changes in the auditory stimuli. For instance, studies by Mager et al. (2005) and by Horváth et al. (2009) found no difference in the MMN in different age groups in adult participants. In contrast, in later ERP components including the P3a, amplitude and latency differences between the age groups were reported. A study by Berti et al. (2013) fits with this perspective: On the one hand, the graphical display of the MMN showed differences between the age groups but this difference was not statistically significant. On the other hand, in the Berti et al. (2013) study no significant aging effect on the P3a was observed. Two aspects are worth noting in this context: First, the largest P3a was observed in the middle age group (39–45 years) of the study (the other two groups had an age range of 18–27 and 59–66 years, respectively). Second, the authors presented also scatterplots summarizing individual ERP amplitudes: For the P3a, the scatterplot suggested a decreased variability in P3a amplitude with increasing age (see Figure 3C in Berti et al., 2013). In addition, Getzmann et al. (2013) demonstrated that aging effects differ significantly depending on the functional state of healthy elderly participants. In other words, high performing older people seem to be more similar to younger participants than low performing elderly participants. Finally, a study by Correa-Jaraba et al. (2016) compared deviant and novelty processing in three age groups (21–29, 51–64, and 65–84 years) and confirmed aging effects on the level of the ERPs only to novel but not to deviant stimuli. Taken together, the picture of aging effects on automatic change detection is less consistent than the initial research in this area had suggested.

### Sources of variability between studies

There are several potential reasons for such an inconsistent pattern of results in aging effects. For instance, a typical source of variability is the actual selection of participants. This may apply to the variation of age range between the different studies as well as to a potential cohort effect between the earliest and the most recent studies within the 20 years range of publication time. For instance, in Germany there is an increasing understanding of the protective mechanisms of social, physical, and intellectual activities on mental health and a 60 year old person in 2017 may not be comparable with a 60 year old person in 1997 with regard to his or her activity levels. Therefore, it is rather unlikely that we will unravel “the” general aging effect based on group comparisons including participants aged between 50 and 85 years investigated between the late 1990th and the late 2010th years. In contrast, another approach to the investigation of aging effects is to focus on the durable invariants of neuro-cognitive processing over the lifespan instead of reaching for the variabilities. This perspective would be fair in the sense that it does not imply the assumption of decreased or disordered processing in healthy aging. More important, the applied experimental paradigm itself constitutes a major source of variability: On the one hand, it is a reasonable approach to apply an experimental protocol, which is well-established in laboratory cognitive and neuroscientific research, to the investigation of aging effects. On the other hand, however, this particular protocol was typically established with young adults as (standard) experimental participants. In other words, it was optimal for only a fraction of adults, i.e., younger ones who are willing to perform an artificial experimental task. With this, the question arises whether this test was fair to healthy adults in their 5th decade and older. From this perspective, it might not come as a surprise that participants who do not constitute the standard experimental participant will perform differently compared with the standard experimental participant. In contrast, applying an ecologically valid task may result in different effects, for instance, for the reason that even elderly healthy adults have more opportunities to “practice” the task in everyday life. This, again, would be a fair test of performance (and underlying neuro-cognitive processes) of a well mastered task situation. Finally, using the well-established auditory oddball paradigm (or a variation of this paradigm) may run into one specific problem. Using a 2-stimulus (i.e., standard vs. deviant tones) oddball stimulation with fast presentation rate (e.g., in passive protocols with 500 ms SOA) may depict a mixture of different processing steps in the ERP, namely the deviance based and the transient or novelty based modus of change detection (Berti, 2013; Lange et al., 2015; Barceló and Cooper, 2017). This is important because one may assume that the amount of OR responses elicited by deviants in a fast oddball stimulation may vary from trial to trial and that the elicitation of an OR by deviants may differ between young and old adults. For instance, one may assume that the susceptibility of OR triggering by deviants increases with age. In other words, the proportion of deviants that accidently trigger an OR may increase with age. In this case, age would affect the respective ERP correlates and could add significant variability to the frontal P3.

### The present study

The aim of the present study was to test whether the OR differs between two different age groups, namely participants aged in the third and fourth decade of life and participants within the sixth decade of life. In order to test this question, we applied an auditory stimulation with a frequent tone and rare novel stimuli. To prevent tapping overlapping responses of OR and deviance detection, we presented these stimuli with long inter-stimulus intervals (10 s). With this, we expect that novels elicit an OR in participants of both age groups. In addition, for comparison with studies applying a classical oddball stimulation we added also conditions with faster presentation rates (3, 1, and 0.5 s). This allows for evaluating the effect of the presentation rate on the deployment of the OR. In addition, no task was related to the auditory stimulation in order to tap automatic change detection.

With this experimental setting, different outcomes are possible. First, as mentioned above, we expect that rare, novel stimuli (i.e., with slow presentation rate) will elicit an OR as indicated by a frontal P3 (i.e., nP3) and an SCR. Second, studies on aging effects on automatic change detection do not suggest that this function gets lost in healthy aging. Therefore, we expect that both age groups show these indices of an OR. A third aspect is the question whether the OR differs between the two age groups. On the one hand, most studies suggested a decline of automatic change detection (mirrored for instance in diminished ERP correlates of automatic processing of and attention switching to a deviant). On the other hand, these studies were not optimized for eliciting an OR: Presentation rates were faster (typically between 500 ms and 3 s) and deviants instead of novels were presented. Therefore, two patterns of results are conceivable. The first possible outcome is that in each condition, the OR in the elderly adults group is smaller compared with the younger adults group. This outcome would support the notion of a general decline of the functions underlying automatic change detection. The second possible outcome is that there are no differences in the effects of the novels in the different conditions between the two age groups. In other words, there would be no support of age related changes in automatic change detection in the present groups of participants. The third possible outcome is that age related differences are confined only to some of the conditions. For instance, it is likely that younger participants deploy a more efficient processing with faster presentation rates but that this advantage will become less prominent with slower presentation rates. In this case, we would expect that group differences would be obtained in the condition with the fastest presentation rate while in the condition with the slowest presentation rate no group differences should be obtained. This pattern of results would suggest a more complex picture of age effects compared to the idea of a continuous and general sensory and cognitive decline over the lifespan.

## Materials and methods

### Participants

In total, 43 participants in the age range between 18 and 76 years were examined in the present study. This study was carried out in accordance with the recommendations of the general Ethical Code of the German Society of Psychology (DGPs). All subjects gave written informed consent in accordance with the Declaration of Helsinki prior to the experimental session after the nature of the study was explained to them. Twenty-five of the participants were female and all but three participants were right-handed. The participants were screened for their health status and all participants reported to be healthy and free of neurological diseases as well as free from sensory deficits other than a corrected ametropia (i.e., myopia or hyperopia). In particular, the participants were asked to report any hearing deficits (e.g., tinnitus, anacusis) they were aware of and whether they use a hearing aid. Only applicants with a good health status and without any known hearing deficits were included. The participants were either recruited at the university from students of different disciplines or they were recruited outside of the university by individual approach in friends and families of the project members. The university students were planned to serve as a young adults group. In contrast, the participants recruited outside of the university were designated for the older adults group and, therefore, in this population people older than 50 years were mainly approached. With regard to their age, participants were either assigned to the younger or to the older adults group with a (theoretical) cut-off point at 50 years. In addition, five participants were excluded from further data analysis either because of too low data quality due to technical problems or high number of artifacts like eye-movements (four participants; age range: 23–32 years) or because of hearing deficits (i.e., reporting to need a hearing aid: one participant, 76 years). This resulted in two groups with 19 participants in each group.

Mean age of the participants in the younger adults group was 23.7 years and the age range was 19 to 38 years. All but three participants were right handed and 15 of the participants were female. Due to the age range, this group will be referred to as the 19–38 years group.

Mean age of the participants in the older adults group was 63 years and the age range was 55–72 years. All participants were right handed and 10 of the participants were female. Due to the age range, this group will be referred to as the 55–72 years group.

The participants of the 55–72 years group underwent additional screening of their health status and a further short experimental procedure to test for their ability to process auditory stimuli in the relevant frequency range (procedure described below). To evaluate the health status of the participants of the 55–72 years group, first, participants were interviewed about any health issues or medications and second, we assessed the Instrumental Activity of Daily Living (IADL) scale (Lawton and Brody, 1969). We applied the analysis of the original IADL as proposed by Kalbe et al. (2005), by calculating a sum score ranging from 8 to 31 with 8 referring to “no problems at all” and 31 referring to “severe disabilities.”

With two exceptions, the participants of the 55–72 years group rated their physical health as good or at least intermediate, including good to intermediate hearing and vision, good to intermediate concentration, and a good to intermediate sleep quality. After further debriefing of the two participants reporting a bad health status, the participants were still included into further data analysis because their reported health issues were neither neurological nor did they affect sensory processing. With regard to drugs affecting the neural or sensory systems, all participants in this group were medication free. The participants of the 55–72 years group reported also high functioning of daily activity as mirrored in the scores of the IADL scale (Lawton and Brody, 1969; Kalbe et al., 2005). All participants but one reported a score of 8 and one participant received a score of 10. This mirrors that all participants of the 55–72 years group were free from any signs of impairment in their daily activity.

Finally, the participants of the 55–72 years group performed a short auditory discrimination test to assure that they had no hearing impairments in the relevant auditory frequency range and no problems to understand task instructions. The task of the participants was a simple response task: 600 Hz sinusoidal tones of 200 ms duration were presented binaurally via headphones. The participants' task was to press a button whenever they perceived a tone. In 40 trials the tone was presented with a sound pressure level of 70 dB; these trials were regarded as reference trials. In addition, tones with different sound pressure levels were randomly mixed within these reference trials. In detail, tones with increased (15 or 25%) and decreased (15 or 25%) SPL were presented in 20 trials each. Finally, 20 catch trials were randomly interspersed in which no tone was presented at all. All participants performed well in these different trials with hit rates ranging between 1 and 0.93.

### Stimuli and experimental procedure

Two types of stimuli were applied in the present study: frequent and rare stimuli. The frequent stimulus was a 600 Hz sinusoidal tone of 200 ms duration (including 5 ms rise and fall time). This stimulus was presented in 90% of the trials during the experiment. Therefore, this stimulus served as the standard stimulus and trials in which the 600 Hz tone was presented are referred to as standard trials. The rare stimulus class contained 80 so-called novels (see Escera et al., 1998): Novels are short environmental sounds with 200 ms duration (including 10 ms rise and fall times) which were only presented once in the experiment. In 10% of the trials, a novel was presented and the occurrence of a novel could not be predicted by the participants; these trials are referred to as novel trials. The stimulus sequence was predefined in a pseudo-randomized order with the constraints that in minimum the initial five trials were standard trials and that at least five standard trials separated two novel trials. All stimuli were presented binaurally via headphones with a sound pressure level of 70 dB in a sound attenuated booth.

The participants completed four different conditions of the oddball stimulation which differed with regard to the inter-stimulus interval (ISI). In short, the ISIs ranged from 10 s to 500 ms and with regard to these four pre-defined ISI levels the conditions are referred to as the 10, 3, 1, and 0.5-s conditions, respectively. In detail, the actual ISIs were randomly jittered around these four time periods resulting in the following mean ISIs per condition: 10,035 ms (range 8,000–12,000 ms jittered in 500 ms steps), 2,948 ms (range 2,400–3,600 ms jittered in 150 ms steps), 998 ms (range 800–1200 ms jittered in 100 ms steps), and 494 ms (range 400–600 ms jittered in 100 ms steps). Each condition consisted of 200 trials (including 20 novel sounds) which were realized in one block per condition except for the 10-s condition which was split into two blocks.

The stimulation and the data acquisition took place in a sound attenuated and electrically shielded booth. The participants were instructed to passively listen to the stimulus presentation and they were informed that there was no additional task associated with the tones. Instead, the participants were allowed to read in a self-chosen book during the experiment. During the EEG experiment, the participants sat in a reclining seat and they were asked to pick a position in which they could comfortably sit during each block without the necessity to move. To minimize artifacts, they were asked to avoid every postural movement during the EEG recording. The participants were also informed about the different lengths of the different blocks. After each block, the participants were allowed to take a short break when necessary (for instance, to correct their position or to stretch). Finally, the sequence of the four conditions was varied between the participants in pseudorandomized way to avoid sequence effects. In more detail, eight different sequences of the ISI conditions were programmed in which each ISI condition served as starting block twice and the detailed succession of the ISI conditions was changed within each sequence.

### SCR recording and analysis

Skin conductance was measured by a constant voltage system (0.5 V) using a bipolar recording with two Ag/AgCl electrodes (0.8 cm diameter) filled with 0.05 M NaCl electrolyte. The electrodes were attached to the thenar and hypothenar eminences of the left hand. Skin conductance was digitized at 10 Hz and stored on an IBM PC for offline analysis.

In order to determine the amplitude of stimulus-related skin conductance responses that overlap substantially especially for short ISIs, we used a continuous decomposition analysis as implemented in Ledalab 3.4.9 (Benedek and Kaernbach, 2010). In short, this approach involves a deconvolution of the recorded electrodermal data, which separates it into continuous signals of tonic and phasic activity. From the phasic activity, we determined the average driver for a response window of 0.5 to 3 s after stimulus onset. The minimum amplitude threshold criterion was set to 0.01 μS. These values were log-transformed using the natural logarithm to reduce the skew of the amplitude distribution (Venables and Christie, 1980). In order to avoid large overlap between SCRs to novel sounds with subsequent standard stimuli, we excluded the SCRs to standards directly following novel stimuli from all analyses. Moreover, SCRs were excluded from further analysis when artifacts were detected in the EEG recordings of the respective trial (see below). Finally, SCR amplitudes were separately averaged for novel and standard stimuli within each ISI condition.

### EEG recording and analysis

#### EEG recording

The EEG was recorded using a Synamps amplifier (Neuroscan, Virginia, USA) from seven cap-mounted electrodes (Easy-Cap, FMS, Munich, Germany) of the 10–20 system (F3, Fz, F4, Cz, Pz, O1, and O2) plus the left and the right mastoid; the reference electrode was placed on the tip of the nose and the ground electrode was placed at FPz. The EEG was recorded with a sampling rate of 250 Hz with an online 0.05–50 Hz band-pass filter. In addition, the vertical and horizontal electrooculogram (EOG) was recorded to control for eye-movements.

#### Computation of the ERPs

The EEG was filtered offline with a 1–30 Hz band-pass and artifacts were removed from further analysis by excluding epochs with high activity in the EOG channels (i.e., whenever the standard deviation within a 200 ms interval exceeded 60 μV). ERPs were computed for standard and novel stimuli separately for the four ISI conditions. Similar to the electrodermal data, standard trials directly following novel sounds were discarded from the analyses. The ERPs were averaged within a time window from 200 to 500 ms relative to stimulus onset; the 200-ms pre-stimulus interval served as a baseline. In the 55–72 years group, the average number of epochs included in the ERP computation varied between 113 (3-s ISI) and 120 (1-s ISI) for standards and between 14 (0.5-s ISI) and 15 (10-s ISI) for novels; in the 19–38 years group, the average number of EEG epochs varied between 109 (0.5-s ISI) and 113 (10-s ISI and 3-s ISI) for standards and between 14 (0.5-s ISI) and 15 (10-s ISI) for novels. No systematic differences in the number of valid trials between the two groups and the different ISI conditions were revealed by statistical analyses with two mixed-effects 2 (*Age group*) × 4 (*ISI*) analyses of variance (ANOVAs): standards: *Age group F*_(1, 36)_ < 1; *ISI F*_(3, 108)_ < 1; *Age group* × *ISI F*_(3, 108)_ = 1.21, *p* = 0.311; novels: *Age group F*_(1, 36)_ < 1; *ISI F*_(3, 108)_ = 1.55, *p* = 0.206; *Age group* × *ISI F*_(3, 108)_ < 1; all ${\eta^{2}}_{p}$ < 0.05. To further increase the signal-to-noise ratio, the ERPs were filtered with a 10 Hz low-pass. These ERPs served as basis for further analysis.

#### Computation of global field power

In a first step, the global field power (GFP) of the ERPs were analyzed in order to identify micro-states (see Lehmann and Skrandies, 1980; Hamburger and van der Burgt, 1991; Michel et al., 1993) of the processing of frequent and rare stimuli. The GFP depicts a single measure of global response strength of the ERP and displays the standard deviation of all electrodes at a given point in time (for the formula see Brunet et al., 2011) measured in μV. We applied the GFP to distinct information processing steps (the so-called micro-states; see Lehmann and Skrandies, 1980; Murray et al., 2008) which are indicated by the curve progression (i.e., distinct peaks in the GFP). This was done (1) for identification of relevant time-windows for the ERP analysis and (2) to test for group differences in global neuro-cognitive processing. The identification of functionally distinct time-windows was based on visual evaluation of the mean GFP in frequent and rare stimuli in the 10-s ISI condition of both age groups because these GFPs depicted the strongest activity in the EEG data. This evaluation indicated three distinct time-windows ranging from 70 to 160 ms, 160 to 250 ms, and 250 to 450 ms. Due to differences in the signal-to-noise ratio, the GFP data was baseline-corrected before statistical analysis and for graphical display.

### Statistical analysis of the psychophysiological data

All statistical analyses were computed using the *base* and the *stats* packages of the R software package (version 3.3.1; R Core Team, 2014). To test for group differences and for effects of the repeated-measure factors *Stimulus type* and *ISI condition*, a series of mixed-effects analyses of variance (ANOVA) were computed (details of the tested models are reported in the following sections). For all statistical tests, the α-level was set to 5% such that *p* < 0.05 was regarded as statistically significant. In addition, for all ANOVAs partial Eta squared ${\eta^{2}}_{p}$ was calculated as a measure of effect size. In addition, for the analysis of the SCR and the EEG data, the Greenhous-Geisser correction was applied wherever the degrees-of-freedom in the numerator were >1. In this case, we report uncorrected degrees-of-freedom, Greenhouse-Geisser epsilon (ε), and corrected *p*-values.

#### SCR analysis

To determine group differences in electrodermal responding, we conducted a mixed-effects ANOVA with the between-group factor *Age group* (2 levels) and the within-group factors *Stimulus type* (2 levels) and *ISI* (4 levels) on the log-transformed SCR responses.

#### GFP analysis

In order to depict group differences, the GFP was averaged in the three identified time-windows (i.e., 70–160, 160–250, and 250–450 ms) separately for the trial types in the different conditions. The mean GFP was then analyzed by a mixed-effects ANOVA with the between-group factor *Age group* (2 levels) and the within-group factors *Stimulus type* (2 levels), *ISI* (4 levels), and *Time window* (3 levels).

#### ERP analysis

The analysis of the ERPs focused on the three time windows identified by the GFP analysis. With regard to the hypotheses, for each of the three midline electrodes (Fz, Cz, and Pz) the ERPs were averaged within the respective time windows. Three mixed-model ANOVAs with the *Age group* (2 levels) and the within-group factors *Stimulus type* (2 levels), *ISI* (4 levels), and *Electrode* (3 levels) were computed. In case of significant interactions of some factors indicating that the effect of either *Stimulus type* or *ISI* is modulated by the group factor, subsequent mixed-effects ANOVAs at Fz separately for the three time windows were computed. We confined the *post-hoc* analysis to the ERP amplitudes at the Fz electrode because the change detection and processing related ERPs typically show a frontal distribution. We conducted further *post-hoc* analyses by computing statistical tests for the difference waves at Fz. This allows for a more detailed analysis of the change detection related ERP components.

### Post-experimental debriefing

After finishing the EEG-recording, all participants were debriefed about their experience during the experiment. For instance, they were asked whether they heard the stimuli, what kind of stimuli they heard, and to rate whether they were distracted from reading their book by the parallel auditory stimulation. To this end the participants used a 7 point scale ranging from −3 (stimuli were distracting) to 3 (stimuli were unnoticed) and rated each of the four ISI conditions separately with regard to this scale. Due to technical problems, three ratings from the 19–38 years group were lost; therefore, the ratings in this group are based on 16 participants. An *Age group* × *ISI* ANOVA yielded a significant effect of the factor ISI on the subjective ratings of the auditory stimuli but no other statistically significant effects: *Age group F*_(1, 34)_ = 3.93, *p* = 0.056, ${\eta^{2}}_{p}$ = 0.10; *ISI F*_(3, 102)_ = 10.67, *p* < 0.001, ${\eta^{2}}_{p}$ = 0.24; *Age group* × *ISI F*_(3, 102)_ = 2.55, *p* = 0.059, ${\eta^{2}}_{p}$ = 0.07. Subjectively perceived distraction by auditory stimuli increased monotonically with shorter ISIs: 0.5-s ISI −0.93, 1-s ISI −0.44, 3-s ISI 0.11, 10-s ISI 0.25.

## Results

**Figure 1:**
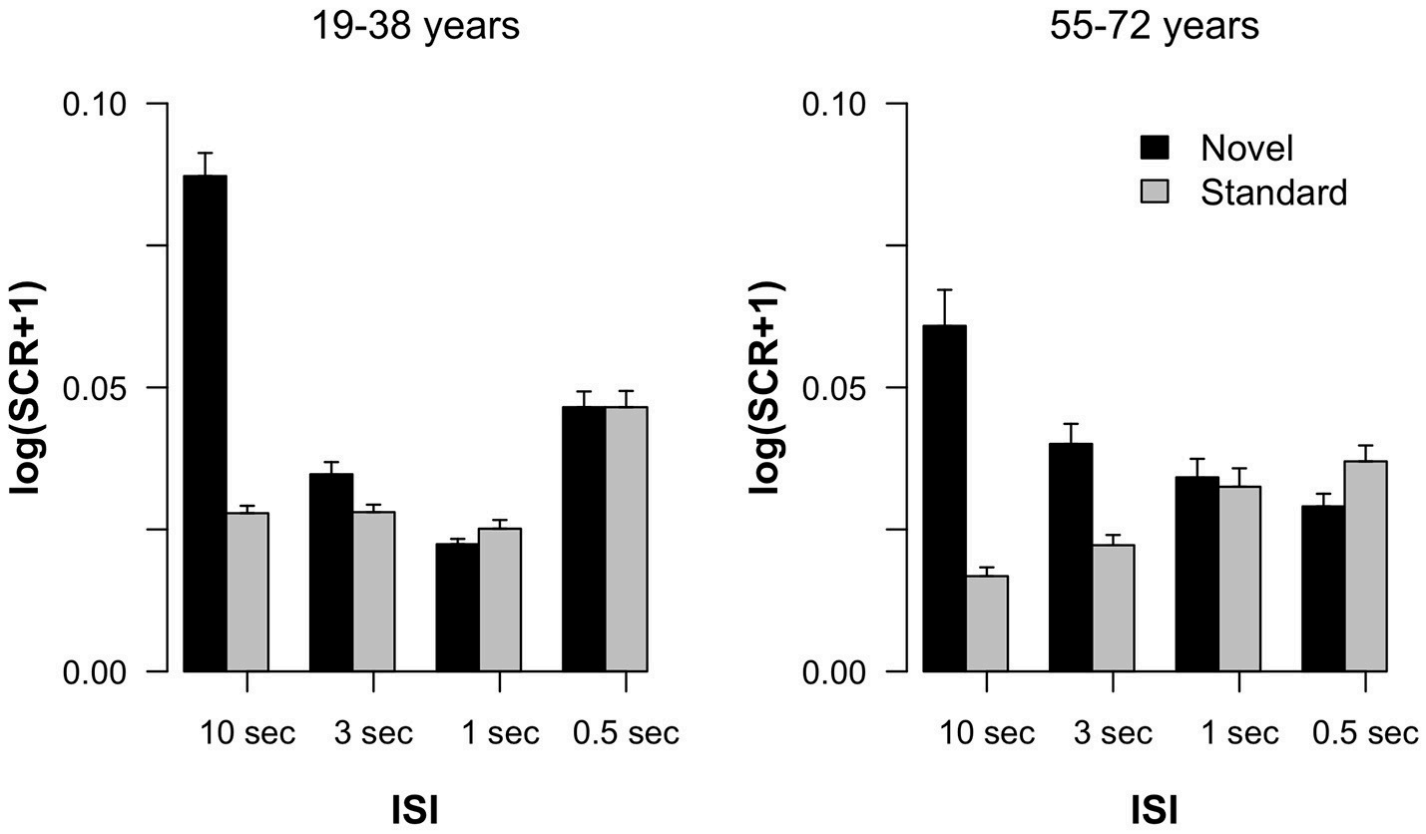
Log-transformed skin conductance response amplitudes in standard and novel trials as a function of ISI, separated for the two groups of participants. Error bars indicate standard errors of the mean.

Figure 1 summarizes the SCR results obtained in the experiment. In both age groups, a pronounced SCR was elicited by novels in the 10-s ISI condition suggesting the elicitation of stable ORs in this trial type. In the 10-s condition the difference between SCRs elicited by standard and by novel stimuli was also most pronounced. In addition, a difference in standard and novel SCRs was observable in the 3-s condition, too, but it declined with shorter ISIs. The *Age group* × *Stimulus type* × *ISI* ANOVA revealed main effects of *Stimulus type F*_(1, 36)_ = 12.84, *p* < 0.001, ${\eta^{2}}_{p}$ = 0.26, and *ISI F*_(3, 108)_ = 8.05, ε = 0.70, *p* < 0.001, ${\eta^{2}}_{p}$ = 0.18, as well as an interaction of both factors *F*_(3, 108)_ = 13.88, ε = 0.54, *p* < 0.001, ${\eta^{2}}_{p}$ = 0.28. All other effects failed to reach statistical significance: *Age group F*_(1, 36)_ < 1; *Age group* × *Stimulus type F*_(1, 36)_ < 1; *Age group* × *ISI F*_(3, 108)_ = 2.10, ε = 0.70, *p* = 0.13, ${\eta^{2}}_{p}$ = 0.06; *Age group* × *Stimulus type* × ISI *F*_(3, 108)_ = 1.59, ε = 0.54, *p* = 0.22, ${\eta^{2}}_{p}$ = 0.04. To follow up on the *Stimulus type* × *ISI* interaction, we calculated pairwise *t*-tests between SCRs to novel and standard stimuli within each ISI condition across groups. These analyses revealed statistically significant differences for the 10-s *t*_(37)_ = 4.10, *p* < 0.001, Cohen's *d* = 0.67, and 3-s condition *t*_(37)_ = 2.75, *p* = 0.009, *d* = 0.45, but not for the 1-s *t*_(37)_ = 1.83, *p* = 0.08, *d* = 0.30, or 0.5-s condition *t*_(37)_ < 1.

**Figure 2:**
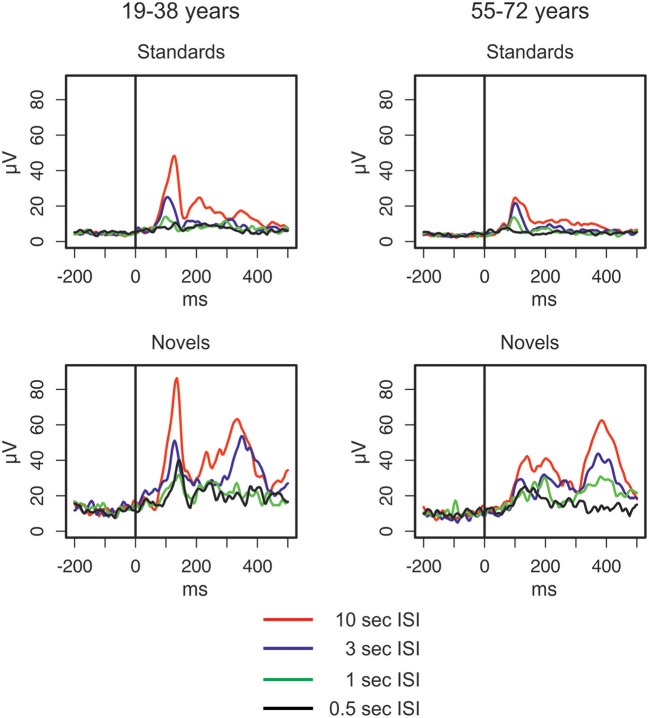
Global field power elicited by standard and novel auditory stimuli separated for the two groups of participants and the four different ISI conditions (for details see main text).

The GFP (see Figure 2) shows differences between standard and novel trials, the four ISI lengths, and the two age groups. In the longest ISI condition, a pronounced early peak (around 100 ms) was observable in both age groups in standard and novel trials. Depending on the trial type and condition, two subsequent peaks were observable around 200 ms and around 350 ms. To sum up, the strongest changes in the GFP were found for novel stimuli and younger participants showed a trend to more pronounced GFP activity compared with older participants. This was also reflected in the statistical analyses (see Table 1) which showed main effects of *Age group* and *Stimulus type*. In addition, also the factors *Time window* and *ISI* showed main effects. Even though a pronounced difference between the two age groups was observable in the early peak of standard and novel GFP in the 10-s ISI condition, the factor Age group did not reveal statistically significant interactions in the four-way ANOVA. The only significant interactions were two-way interactions between *Stimulus type* and *ISI* and the factor *Time window*.

**Table 1:** Statistical evaluation of the group effect (19–39 vs. 55–78 years) and the effects of the repeated-measure factors Stimulus type (standard vs. novel), ISI (10, 5, 3, or 0.5-s ISI condition), and Time window (70–160, 160–250, or 250–450 ms) on the global field power by means of analysis of variance (ANOVA).

	**df**	***F***	**ε**	**${\eta^{2}}_{p}$**
Age group (A)	1, 36	7.57^*^	–	0.17
ISI (I)	3, 108	27.06^***^	0.84	0.43
Stimulus type (S)	1, 36	272.31^***^	–	0.88
Time window (T)	2, 72	161.79^***^	0.59	0.82
A × I	3, 108	2.19	0.84	0.06
A × S	1, 36	2.82	–	0.07
A × T	2, 72	2.95	0.59	0.08
I × S	3, 108	1.64	0.91	0.04
A × I × S	3, 108	1.51	0.91	0.04
I × T	6, 216	43.67^***^	0.61	0.55
A × I × T	6, 216	2.95	0.61	0.05
S × T	2, 72	48.75^***^	0.64	0.58
A × S × T	2, 72	0.74	0.64	0.02
I × S × T	6, 216	1.42	0.70	0.04
A × I × S × T	6, 216	0.53	0.70	0.01

F-values, Greenhouse-Geisser ε, uncorrected degrees of freedom (df), and partial eta-squared ($\eta_{p}^{\text{2}}$) are summarized; corrected significance levels:

* *p < 0.05*,

*** *p < 0.001*.

**Figure 3:**
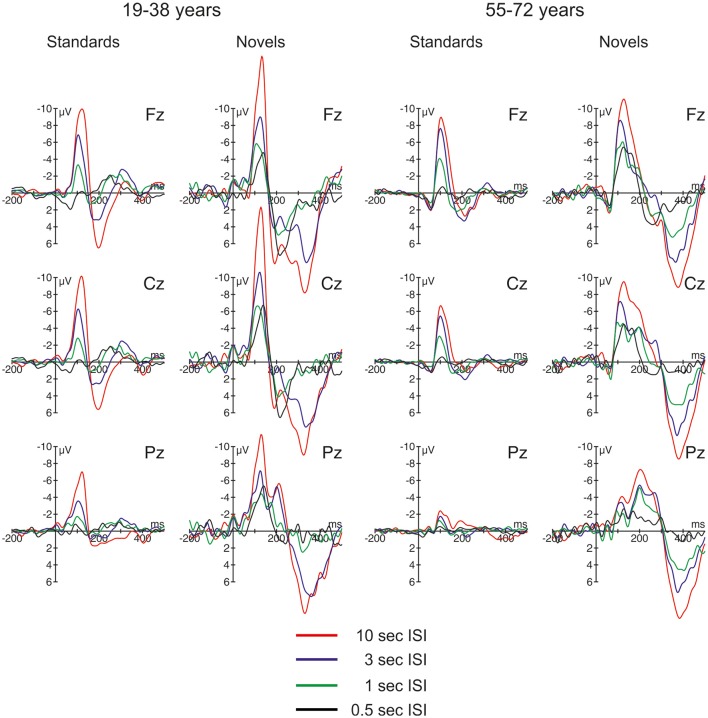
Overview of the event-related brain potentials (ERPs) elicited at midline electrodes by standard and novel auditory stimuli separated for the two groups of participants and the four different ISI conditions.

**Figure 4:**
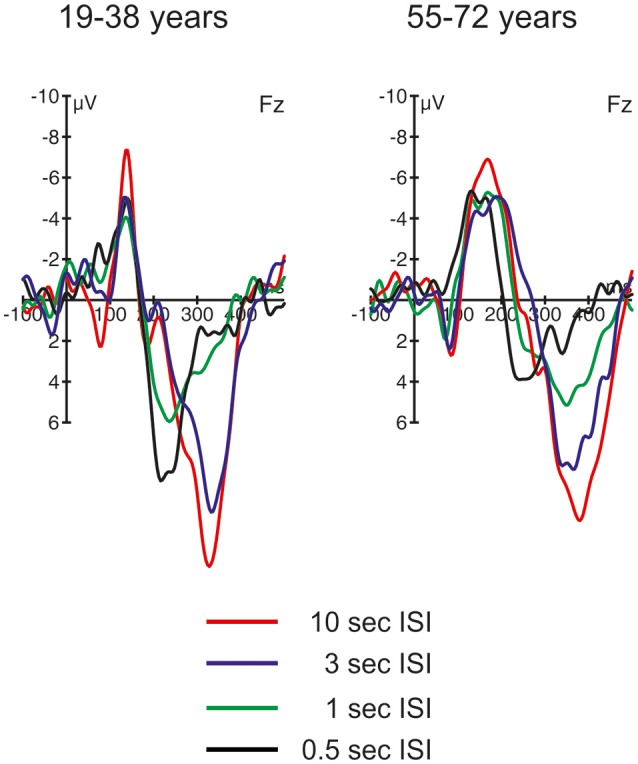
ERP difference waves (i.e., ERPs elicited by novel stimuli minus ERPs elicited by standard stimuli) at Fz in the four different ISI conditions, separated for the two groups of participants.

The ERPs are summarized for the midline electrodes in Figure 3, the respective difference waves are depicted in Figure 4 at Fz. Especially the ERPs elicited by novel stimuli mirror the temporal progression of the brain waves with a prominent initial negative peak around 100 ms followed by two positive deflections around 200 and 300 ms. However, this sequence was modulated by the ISI: While the second positive deflection was most pronounced in the two longest ISI conditions, the first positive deflection increased with decreasing ISI duration. In general, the three-phase sequence for novel stimuli was observable in both age groups with the remarkable difference that in the 1-s ISI condition the participants in the 55–72 years group showed a stronger second positive deflection compared with the 19–38 years group. In standard stimuli, the early negative peak was also pronounced (and in the 10-s condition only slightly smaller in standards compared with novels) and the first positive peak was observable in the longer ISI conditions, too. In contrast, the second positivity was not observable in standard stimuli irrespective of ISI duration or age group. Regarding the timing and polarity of the three peaks, this sequence can be identified as the N1, the P3a, and the nP3 component.

Table 2 summarizes the statistical analysis of the effects of the factors *Stimulus type, Age group, ISI*, and *Electrode* separately for the three identified time windows. This analysis depicted a comparable variety of effects and interaction of these factors. Noteworthy, only *Stimulus type* and *Electrode* revealed main effects within all three time windows. This reflects that the relevant ERP components are best depicted in the difference waves of novels and standard stimuli and are typically observed at frontal electrodes. Therefore, three subsequent three-way ANOVAs were computed separately for the average amplitude at Fz to test for group effects as well as the effect of the *Stimulus type* and the *ISI* condition. In the 70–160 ms time window, the ANOVA yielded only main effects of *Stimulus type* and *ISI*: *Age group F*_(1, 36)_ < 1; *Stimulus type F*_(1, 36)_ = 53.28, *p* < 0.001, ${\eta^{2}}_{p}$ = 0.60; *ISI F*_(3, 108)_ = 81.01, ε = 0.60, *p* < 0.001, ${\eta^{2}}_{p}$ = 0.69; *Age group* × *Stimulus type F*_(1, 36)_ < 1; *Age group* × *ISI F*_(3, 108)_ = 2.41, ε = 0.60, *p* = 0.102, ${\eta^{2}}_{p}$ = 0.06; *Stimulus type* × *ISI F*_(3, 108)_ = 2.04, ε = 0.83, *p* = 0.13, ${\eta^{2}}_{p}$ = 0.05; three-way interaction *F*_(3, 108)_ < 1. In the 160 to 250 ms time window, only the main effect of the factor *Stimulus type* and the three-way interaction revealed no significant effects: *Age group F*_(1, 36)_ = 12.82, *p* = 0.001, ${\eta^{2}}_{p}$ = 0.26; *Stimulus type F*_(1, 36)_ < 1; *ISI F*_(3, 108)_ = 6.04, ε = 0.57, *p* = 0.003, ${\eta^{2}}_{p}$ = 0.14; *Age group* × *Stimulus type F*_(1, 36)_ = 28.43, *p* < 0.001, ${\eta^{2}}_{p}$ = 0.44; *Age group* × *ISI F*_(3, 108)_ = 9.68, ε = 0.72, *p* < 0.001, ${\eta^{2}}_{p}$ = 0.21; *Stimulus type* × *ISI F*_(3, 108)_ = 11.85, ε = 0.83, *p* < 0.001, ${\eta^{2}}_{p}$ = 0.25; three-way interaction *F*_(3, 108)_ < 1. Finally, in the 250–450 ms time window, no group effect nor interactions with the group factor were obtained: *Age group F*_(1, 36)_ = 2.88, *p* = 0.10, ${\eta^{2}}_{p}$ = 0.07; *Stimulus type F*_(1, 36)_ = 120.21, *p* < 0.001, ${\eta^{2}}_{p}$ = 0.77; *ISI F*_(3, 108)_ = 29.80, ε = 0.77, *p* < 0.001, ${\eta^{2}}_{p}$ = 0.45; *Age group* × *Stimulus type F*_(1, 36)_ < 1; *Age group* × *ISI F*_(3, 108)_ = 2.30, ε = 0.77, *p* = 0.099, ${\eta^{2}}_{p}$ = 0.06; *Stimulus type* × *ISI F*_(3, 108)_ = 27.66, ε = 0.91, *p* < 0.001, ${\eta^{2}}_{p}$ = 0.43; three-way interaction *F*_(3, 108)_ = 1.27, ε = 0.91, *p* = 0.29, ${\eta^{2}}_{p}$ = 0.03.

**Table 2:** Statistical evaluation of the group effect (19–39 vs. 55–78 years) and the effects of the repeated-measure factors Stimulus type (standard vs. novel), ISI (10, 5, 3, or 0.5-s ISI condition), and Channel (Fz, Cz, or Pz) of the average ERP amplitude within the 70–160 ms, the 160–250 ms, and the 250–450 ms time window by means of separate analyses of variance (ANOVA).

	**df**	**70–160 ms**			**160–250 ms**			**250–450 ms**		
		***F***	**ε**	**${\eta^{2}}_{p}$**	***F***	**ε**	**${\eta^{2}}_{p}$**	***F***	**ε**	**${\eta^{2}}_{p}$**
Age group (A)	1, 36	4.43^*^	–	0.11	15.45^***^	–	0.30	0.27	–	0.01
Electrode (E)	2, 72	23.03^***^	0.59	0.39	33.68^***^	0.61	0.48	9.68^**^	0.68	0.21
Stimulus type (S)	1, 36	70.80^***^	–	0.66	9.80^**^	–	0.21	93.40^***^	–	0.72
ISI (I)	3, 108	62.05^***^	0.64	0.63	0.39	0.74	0.01	33.34^***^	0.76	0.48
A × E	2, 72	6.04^*^	0.59	0.14	4.18^*^	0.61	0.10	11.99^***^	0.68	0.25
A × S	1, 36	3.23	–	0.08	20.71^***^	–	0.37	0.28	–	0.01
A × I	3, 108	4.28^*^	0.64	0.11	8.22^***^	0.74	0.19	2.57	0.76	0.07
E × S	2, 72	7.69^**^	0.78	0.18	25.65^***^	0.60	0.42	26.44^***^	0.60	0.42
A × E × S	2, 72	4.52^*^	0.78	0.11	14.60^***^	0.60	0.29	5.04^*^	0.60	0.12
E × I	6, 216	32.56^***^	0.40	0.47	27.93^***^	0.38	0.47	2.72	0.43	0.07
A × E × I	6, 216	0.65	0.40	0.02	7.46^***^	0.38	0.17	3.11^*^	0.43	0.08
S × I	3, 108	0.87	0.82	0.02	16.67^***^	0.85	0.32	29.06^***^	0.88	0.45
A × S × I	3, 108	0.01	0.82	>0.01	0.41	0.85	0.01	2.19	0.88	0.08
E × S × I	6, 216	4.21^**^	0.61	0.10	4.38^**^	0.52	0.12	1.99	0.46	0.05
A × E × S × I	6, 216	1.28	0.61	0.03	2.68^*^	0.52	0.07	2.18	0.46	0.06

F-values, Greenhouse-Geisser ε, uncorrected degrees of freedom (df), and partial eta-squared ($\eta_{p}^{\text{2}}$) are summarized; corrected significance levels:

* *p < 0.05*,

** *p < 0.01*,

*** *p < 0.001*.

Figure 4 depicts the difference waves at Fz. With regard to the ERP components related to automatic change detection and attentional allocation, the difference waves revealed some communalities in the two age groups as well as some differences in the sequence of components between the two age groups. The early negative component peaking between 100 and 200 ms after stimulus onset was sharper and more pronounced in the 19–38 years group compared to the broader component structure in the 55–72 years group. However, the amplitudes were comparable in both age groups and show the most pronounced early negative deflection with the longest ISI while in the other ISI conditions the negative deflections were of comparable strength. The statistical analysis by means of independent *t*-tests depicted no significant group differences in this time window (see Table 3). It is worth noting that this early negative deflection in the 55–72 years group suggests a bi-phasic sequence at least with the 0.5, 1, and 3 s ISIs. In addition, the positive peaks around 300 ms showed similar characteristics in both age groups: The amplitude was maximal with the longest ISI (10 s) and decreased with decreasing ISI duration. Again, the statistical analysis showed no significant group differences (see Table 3). In contrast, the difference waves around 200 ms depicted pronounced differences between the two age groups: Here, the 19–38 years group depicted a marked positive difference in the 0.5 and 1-s ISI conditions but no significant difference between the standard and novel stimuli in the 3-s and 10-s ISI conditions. In the 55–72 years group, however, significant negative amplitudes were obtained for all ISIs but the 0.5-s ISI; in the 0.5-s ISI condition there was a tendency to a positive deflection with a slightly later peak compared to the positive peak in the 19–38 years group. Finally, Table 3 summarizes group mean amplitudes of the obtained difference waves in the three time windows separately for all four ISI conditions.

**Table 3:** Summary of the mean amplitudes (in μV) of the ERP difference waves (novel stimuli minus standard stimuli) at Fz separately for the two age groups, the four ISI conditions and the three time windows.

	**70–160 ms**				**160–250 ms**				**250–450 ms**			
	**19–38 years**		**55–72 years**		**19–38 years**		**55–72 years**		**19–38 years**		**55–72 years**	
	***M***	**95% CI**	***M***	**95% CI**	***M***	**95% CI**	***M***	**95% CI**	***M***	**95% CI**	***M***	**95% CI**
0.5 s	−3.17	−4.83, −1.52	−3.43	−4.61, −2.26	5.49	3.16, 7.84	−0.19	−1.68, 1.30	1.96	0.60, 3.32	1.55	0.63, 2.47
1 s	−2.45	−4.22, −0.68	−2.30	−3.31, −1.30	3.35	1.07, 5.63	−2.77	−4.26, −1.27	1.93	0.58, 3.28	3.36	2.06, 4.66
3 s	−2.16	−3.85, −0.48	−1.50	−2.24, −0.76	0.87	−1.37, 3.10	−3.98	−6.05, −1.92	5.28	4.01, 6.54	4.71	2.89, 6.52
10 s	−2.36	−4.29, −0.42	−1.93	−3.60, −0.26	1.80	−0.93, 4.53	−3.93	−5.81, −2.06	6.24	4.33, 8.15	6.58	4.81, 8.35

*M, averaged amplitude in the respective time window in μV; 95% CI, 95% confidence interval of the respective group mean*.

## Discussion

We presented novel sounds embedded in a sequence of standard stimuli with a slow presentation rate to trigger the OR in passive listeners. The results obtained in the 10-s ISI condition depicted an OR in both age groups mirrored by the elicitation of a pronounced SCR. Novels also elicited pronounced N1 and frontal P3 components in the ERP. Especially the frontal P3 together with the SCR response provided evidence that novels triggered an OR in this condition. The analysis of group differences in this condition, therefore, allows for unravelling whether healthy aging affects the mechanisms underlying the OR. In addition, the pattern of results with variations in the ISI adds additional insight into mechanisms underlying the automatic processing of changes in audition. In the following, we first discuss the results in the 10-s ISI condition, second, we discuss the variations of the results with increasing stimulus presentation rate, and third, we focus on the question how the pattern of results in the frontal P3 can be interpreted regarding the automatic processing of changes.

### Aging effects on the OR: the 10-s ISI condition

In both age groups, the SCR in the 10-s condition increased for novel as compared to standard trials. This was accompanied by increased N1 and nP3 amplitudes to novel stimuli (see the difference waves in Figure 4); these effects showed no group differences. The N1 is an index of the sensory processing of an incoming stimulus and mirrors sensory gating but is also linked to selective attention (for a recent review see Joos et al., 2014). Therefore, the increased N1 mirrors differences in the saliency between standard and novel stimuli. In contrast, the nP3 is a unique correlate of novelty processing and the OR (Lange et al., 2015; Barry et al., 2016) and, therefore, indicates the elicitation of an OR. As noted above, the comparable SCR and nP3 responses to the novels suggest that the OR is effective in both age groups and does not indicate age-related changes in the OR and its underlying mechanisms.

Evaluation of the ERPs in both, novel and standard trials, allows for further conclusions. First, in both age groups a pronounced N1 was elicited by novel and standard stimuli. The strongest N1, however, was observed for novel stimuli in the 19–38 years group. In contrast, the difference between the novel N1 and the standard N1 was not as pronounced in the 55–72 years group. Another remarkable difference between the two groups is that in the younger participants group the N1 was followed by a pronounced P2 for both stimulus types. In contrast, in the older adults group the P2 was smaller or not identifiable. Especially the pronounced P2 for standards in the younger adults group suggests that with the slow presentation rate (average ISI of 10 s) a more effective processing of auditory stimuli is possible. In contrast, the SCRs do not indicate that standards in the 10-s ISI condition elicited an OR in the 19–38 years group. Considering that the nP3 in this condition showed no remarkable difference between the two age groups, it is an interesting outcome that aging effects were observable in comparably early processing steps as reflected by the N1 and the P2 while subsequent processing steps (i.e., mirrored in the nP3) seemed to be less affected by age in healthy adults. With regard to the main question of the present study we can conclude that at least in our sample the OR was similarly effective in both age groups.

### Switching from OR to deviant processing: the 3-s, 1-s, and 0.5-s ISI conditions

With decreasing ISI length the SCR difference between standard and novel stimuli decreased in both age groups, which suggests that with faster presentation rates novels forfeit the capability of triggering an OR. In the difference waves, this is correlated with the decrease in nP3 amplitude. This, again, supports that nP3 is a central-nervous correlate of the OR (see Lange et al., 2015; Barry et al., 2016). However, the development of nP3 and SCR strength differed in the two age groups. While in the 55–72 years group the decline was a gradual one, in the 19–38 years group there was a qualitative change between the 3-s and the two shortest ISI conditions. In other words, in the 55–72 years group the nP3 seemed to decline linearly with decreasing ISI length. In contrast, in the 19–38 years group the pronounced nP3 was elicited only in the 3-s ISI condition while in the 1-s and the 0.5-s ISI condition a pronounced earlier P3 peaking around 250 ms was observable. This earlier, frontal positive peak in the difference waves resembles the P3a, which is assumed to be correlated with different cognitive functions than the nP3 (see Barry et al., 2016). It is worth noting that the P3a was also elicited by novels in the 55–72 years group but only in the shortest ISI condition with the fastest presentation rate (500 ms ISI in average). In contrast, in standard trials the frontal N1 showed a linear decline with decreasing ISI length in both age groups, mirroring the increased habituation to repetitive auditory stimuli with short ISIs. Even though the N1 amplitude differed numerically between the two groups, the general pattern of results does not proof strong aging effects but suggests a comparable neuronal response to standard stimuli.

By comparing the ERP responses at Fz on the rare stimulus class between the different ISI conditions, a clear distinction between two different frontal P3s can be drawn. This resembles the distinction of nP3 and P3a as favored by Barry et al. (2016); see also Lange et al., 2015). Based on this distinction, the frontal ERP effects mirror two different aspects of automatic change detection: an OR with slower presentation rates mirrored in the later nP3 and deviance detection with faster presentation rates mirrored in the earlier P3a. This fits to the interpretation in a study by Berti (2013) who also demonstrated that two different frontal positive components between 200 and 400 ms can be elicited by rare, unexpected stimuli. In this study, the difference was whether the rare auditory event was either embedded within an ongoing auditory stimulation and resembled an oddball in the classical oddball stimulation (i.e., a deviant) or was a rare, sudden onset of an auditory event without an accompanying standard stimulation (i.e., a transient). Berti argued that deviance detection is accompanied by the earlier frontal positivity and transient or novelty detection by the later frontal positivity. This would fit to the present results because it is likely that with increasing presentation rate saliency of novels may decrease. In other words, with faster presentation rate processing of the novel may resemble the processing of deviants and the contribution of unique novelty processing in auditory change detection may decline, too (see Berti, 2012). At present, it remains open whether the results from Berti (2013) fully fit to the nP3/P3a distinction because the earlier positivity peaked around 200 ms and, with this, could also resemble a P2. However, the results from Berti (2013) at least demonstrate how much the context of stimulus presentation modulates the processing of changes in the auditory input; a perspective which fits to the present study, too.

### Summary of the age group differences

Taken together, while the two age groups show highly comparable psychophysiological correlates of the processing of standard and novel sounds, there are also differences suggesting at least some effects of aging on automatic change detection. It is worth noting that these differences were obtained with faster presentation rates because this mirrors earlier findings of aging effects in oddball stimulations (see, for reviews, Kok, 2000; Pekkonen, 2000; Friedman, 2003). For instance, Rimmele et al. (2012) reported a complete lack of a frontal P3 in older adults. The present study, however, suggests that such a finding might be confined to a comparably faster presentation rate because with salient changes (here the novels in the 10 and 3-s ISI conditions) a pronounced frontal P3 was observed. In addition, our results also question whether healthy aging necessarily results in a loss of efficacy of automatic change detection. In our study, at least the efficacy of the OR did not show strong modulations by (healthy) aging. It is important to note that this finding does not imply that there are no aging effects at all because we also tested only a small and presumably special sample of adults in the sixth, seventh, and eighth decade of their lives. For instance, our participants were happy to come to the lab and were highly motivated to perform a comparably boring task just to support scientific understanding of healthy aging. In other words, our participants are not necessarily representative for all adults in this age range. However, within this group of participants and this kind of sensory stimulation we observed highly efficient ORs as one—highly relevant—form of automatic change detection. And even with less strong variants of change detection recent studies demonstrated that aging effects on automatic change detection are not mandatory (see Mager et al., 2005; Horváth et al., 2009; Berti et al., 2013; Getzmann et al., 2013; Correa-Jaraba et al., 2016). At least these studies suggest that we should avoid too strong generalizations about aging effects from one type of (typically non-ecological) laboratory tasks.

## Conclusion

The main question of the present study was whether the OR is affected by healthy aging. Here, we could not find an aging effect on the OR. This does not necessarily come to a surprise considering that the OR-eliciting stimuli depict very salient events. However, it is still worth noting that at least in our sample the consequences of such a salient event results in comparable autonomic and neuronal responses in both age groups. This, in contrast, does not imply that flexible adaptation to this change (i.e., a behavioral response) would be as effective in our experimental group (55–72 years) compared to the control group (19–38 years). However, the novelty processing and OR elicitation was effective in the older and the younger participants. In addition, we also found aging effects in our study: The SCR results together with the ERP effects suggest that with increasing presentation rate a switch from an OR-based to a deviancy-based change detection mechanism took place. With respect to the point in time when this switch was observable the two groups differed. From this, one may assume that in our study aging affected the efficacy of deviance processing compared to novelty processing with an earlier onset of OR processing with increasing age. For the reason that the OR is more directly linked to disruption of ongoing cognitive processing, this might be interpreted as an increased distractibility in older adults.

## Ethics statement

The present study included healthy adult participants who had no problems to understand the scientific background and the nature of the study as well as the study protocol. In accordance with the Declaration of Helsinki as well as with the Ethical Code of the German Society for Psychology (DGPs), all participants gave written informed consent after the nature of the study was explained to them. The local ethical committee does not require formal approvement in such cases (i.e., healthy adults participating voluntarily, full informtion about the nature and the protocol of the study prior to the participation, and written consent).

## Author contributions

SB, GV, and MG devised the experimental design. SB and MG set up the experiment and analyzed the data. SB collected the data. SB, MG, and GV wrote the manuscript.

### Conflict of interest statement

The authors declare that the research was conducted in the absence of any commercial or financial relationships that could be construed as a potential conflict of interest.
